# Hydatid disease – An unusual cause of a breast cyst: Case report

**DOI:** 10.1016/j.ijscr.2020.02.066

**Published:** 2020-03-09

**Authors:** Zkria Atia Shekh

**Affiliations:** Department of General Surgery, Damascus Hospital, Damascus, Syria

**Keywords:** Cystic echinococcosis, Breast, Endemic, Isolated, Rare

## Abstract

**Introduction:**

Breast cysts are common and are usually due to fibrocystic disease of the breast. However, in endemic areas a breast cyst can be due to hydatid disease. This diagnosis is often made after surgical excision. Hydatid cysts, caused by the tapeworm Echinococcus granulosus, are common in endemic areas and usually occur in the liver and lung.

**Case presentation:**

A 23-year old female patient presented with a breast lump fearing cancer. Initial assessment demonstrated a hydatid cyst which proved to be isolated.

**Discussion:**

An isolated hydatid cyst in the breast is very rare, occurring in only 0.27% of all hydatid cases. The diagnosis is important for management and the exclusion of hydatid disease elsewhere in the body. The parasite can be detected serologically. Total excision of the cyst without spillage and benzimidazolic drugs are the management of choice.

**Conclusion:**

In endemic areas of hydatid disease, the possibility that a breast lump might be hydatid in origin needs to be considered.

## Highlights

• Breast is unusual site for echinococcosis.
• How to differentiate a simple breast cyst from a hydatid cyst.
• Multiorgan hydatid cyst is common but an isolated breast hydatid cyst is rare.
• Serological tests for hydatid disease are available.

## 1 Introduction

Hydatid disease is due to infection by the tapeworm Echinococcus granulosus in its larval or cyst stage [1]. The tapeworm lives in canids, which are infected by eating the viscera of sheep that contain hydatid cysts. Scolices, contained in the cysts, adhere to the small intestine of dogs and become adult taenia, which attach to the intestinal wall. Each worm sheds approximately 500 ova into the bowel. The infected ova-containing feces of dogs contaminate grass and farmland, and the ova are ingested by intermediate hosts such as sheep, cattle, pigs, and humans. The ova have chitinous envelopes that are dissolved by gastric juice. The liberated ovum then burrows through the intestinal mucosa and is carried by the portal vein to the liver, lung or other organs like the breast; where it develops into an adult cyst. Hydatid disease is most common in sheep-raising areas, where dogs have access to infected offal. These include South Australia, New Zealand, Africa, Greece, Spain, and the Middle East. Primary hydatid disease of the breast is very rare (about 0.27% of all hydatid cases [2]) and very few cases had been published.

The diagnosis of hydatid disease is based on the findings of an enzyme-linked immunosorbent assay (ELISA) for echinococcal antigens, and results are positive in approximately 85% of infected patients [1]. Total excision of the cyst without spillage and chemotherapy is the treatment of choice. Surgical treatment is curative in all cases [3].

We present a case of an isolated breast cyst in a 23-year-old female. This work has been reported in line with the SCARE criteria [4].

## 2 Case presentation

A 23 year- old female from a rural sheep-raising area in the north of Syria presented with a painless breast mass for three months. Her past medical history was unremarkable and she had no systemic symptoms. On examination there was a firm but mobile mass in the upper outer quadrant of the left breast that moved freely and was mildly tender. Echography demonstrated a well-defined mass (2.2 × 3.8 cm) with a capsule and multiple cystic components, thought to be a hamartoma. The mass was excised intact for histopathology. The cut section showed multiple daughter cysts pathognomic for hydatid cystic disease and microscopic examination confirmed the diagnosis. The patient was discharged on albendazole [1,5,6].

## 3 Discussion

Hydatid disease is endemic in Syria and the organs usually affected are the liver (63%) and lung (25%). The breast can be involved in multi-organ disease but isolated breast hydatid disease is rare and usually presents as a cyst managed by either observation or aspiration. In the present case the patient presented with a lump which was excised for a definitive pathological diagnosis. Triple assessment of any breast mass is important to exclude malignancy [7].

Triple assessment includes history and physical examination, radiological imaging and pathology. In our case the patient's clinical assessment and echography were inconclusive and excision biopsy was performed for a definitive pathological diagnosis. Fine needle aspiration (FNA) was not performed but cytology (FNAc) can on occasion diagnose hydatid cyst disease if hooklets are found within the sample [2]. The features of hydatid cyst fluid are that it is clear with specific gravity 1.005–1.009. Casoni’s intradermal test is 75% sensitive) [5]. If a diagnosis of hydatid disease is made preoperatively, treatment with Albendazole is recommended before surgical intervention [5]. Although FNAc is part of the triple assessment of breast masses it is contra-indicated if the ultrasound examination suggests hydatid. Serum antibody detection is specific and sensitive (80% for liver and 40% for lung) [1]. Abdominal ultrasound and chest X-Ray, or a CT scan, can suggest whether there is hydatid disease in the liver or lungs.

It is important to differentiate between a simple breast cyst and a hydatid cyst because in the latter case spillage must be prevented. Surgery is still the gold standard therapy for hydatid disease of the breast [5], together with medical therapy using albendazole (4-week cycles with drug free intervals of two weeks) as it is the commonly used ovicidal, larvicidal and vermicidal [5].

## 4 Conclusion

Hydatid cyst in the breast is a possible if rare diagnosis in endemic areas, and imaging and serological testing can help to exclude or reveal it.

## Declaration of Competing Interest

The author have no financial and personal relationships with other people or organizations that could inappropriately influence this submission.

## Sources of funding

There is no sources of funding.

## Ethical approval

Study is exempt from ethnical approval in my institution.

Approval is obtained; the patient give agreement for publication.

## Consent

Written informed consent was obtained from the patient for publication of this case report and accompanying images.

## Author contribution

Zkria shekh do everything (take history and physical examination, surgery, and writing this paper).

## Registration of research studies

Name Hydatid disease – an unusual cause of a breast cyst, Case report unique identifying number is: researchregistry5357.

https://www.researchregistry.com/browse-the-registry#home/.

## Guarantor

Zkira shekh.

## Provenance and peer review

Not commissioned, externally peer-reviewed.
